# Toward an early clinical diagnosis of MM2‐type sporadic Creutzfeldt–Jakob disease

**DOI:** 10.1002/acn3.51802

**Published:** 2023-06-06

**Authors:** Zhongyun Chen, Yu Kong, Jing Zhang, Min Chu, Li Liu, Kexin Xie, Yue Cui, Hong Ye, Junjie Li, Lin Wang, Liyong Wu

**Affiliations:** ^1^ Department of Neurology Xuanwu Hospital, Capital Medical University Beijing China

## Abstract

**Objective:**

To assess the proportion of clinically diagnosed MM2‐type sporadic Creutzfeldt–Jakob disease (sCJD) in a Chinese cohort, describe the clinical features of MM2‐cortical (MM2C) and MM2‐thalamic (MM2T) type sCJD to improve the early detection of MM2‐type sCJD.

**Methods:**

A total of 209 patients with sCJD admitted to the Xuanwu Hospital between February 2012 and August 2022 were reviewed. The patients were classified into probable MM2C, MM2T‐type sCJD, and other types of sCJD according to current clinical diagnostic criteria. Clinical and ancillary data were compared between the groups.

**Results:**

Fifty‐one (24.4%) patients were clinically diagnosed with MM2‐type sCJD, of which 44 were diagnosed with MM2C‐type sCJD and 7 with MM2T‐type sCJD. In the absence of RT‐QuIC, 27 (61.3%) patients of MM2C‐type sCJD did not meet the US CDC sCJD criteria for possible sCJD on admission, even though the mean period from onset to admission was 6.0 months. However, all of these patients had cortical hyperintensity on DWI. Compared to the other types of sCJD, MM2C‐type sCJD was associated with slower disease progression and the absence of the typical clinical features of sCJD; the MM2T‐type sCJD group had a higher proportion of males, earlier age of onset, longer duration of disease, and a higher incidence of bilateral thalamic hypometabolism/hypoperfusion.

**Interpretation:**

In the absence of multiple typical sCJD symptoms within 6 months, the presence of cortical hyperintensity on DWI should raise concerns for MM2C‐type sCJD after excluding other etiologies. Bilateral thalamic hypometabolism/hypoperfusion may be more helpful in the clinical diagnosis of MM2T‐type sCJD.

## Introduction

Sporadic Creutzfeldt–Jakob disease (sCJD), a fatal transmissible neurodegenerative disease, is caused by an abnormal infectious isoform of prion protein (PrP^Sc^), which is generated by a conformational change of the normal cellular isoform (PrPC). The genotype (methionine, M or valine, V) at polymorphic codon 129 of the prion protein gene (*PRNP*) and the type (1 or 2) of PrP^Sc^ in the brain are the major determinants of the clinicopathological features of sCJD and permit molecular classification of sCJD, that is, MM1, MM2, MV1, MV2, VV1, or VV2.^1^, ^2^, ^3^

MM1‐type sCJD exhibits a typical clinical course and is the most common sCJD in Caucasians, accounting for approximately 46–60% of cases. On the other hand, MM2‐type sCJD is a rarer molecular subtype with unusual symptoms and prolonged survival and accounts for only 4.5–13.6% of the sCJD cases.^4^, ^5^, ^6^, ^7^, ^8^ MM2‐type sCJD can be further subdivided into the cortical (MM2‐cortical or MM2C) and thalamic (MM2‐thalamic or MM2T) forms, also indicated as sporadic fatal insomnia (sFI). The proportion of 129 MM genotypes in the Chinese sCJD population ranges from 97 to 100%, which also suggests the same proportion of MM1 and MM2‐type sCJD.^9^, ^10^ Due to current autopsy conditions and ethical considerations, the vast majority of Chinese patients with sCJD do not undergo autopsy, which has led to unclear molecular classification. Only one case with a clinical diagnosis of MM2T‐type sCJD has been reported in China so far.^11^ The prevalence of MM2‐type sCJD in China is at present unknown. We hypothesize that the proportion of patients with MM2‐type sCJD is underestimated due to the high incidence of the 129MM genotype in China, a similar issue may also affect Asian countries with a high prevalence of the 129MM genotype.^12^

Current diagnostic criteria for sCJD are most appropriate for MM(V)1, the most common subtype and a rapidly progressive multisystem neurological disorder that leads to mutism after only 2–5 months.^13^, ^14^ As such, these criteria do not adequately take into account the heterogeneity of clinical features associated with other sCJD subtypes, such as MM2‐type sCJD and VV2‐type sCJD.^15^ Furthermore, they often require waiting for the full manifestation of neurological symptoms and signs to formulate the clinical diagnosis of possible sCJD. The clinical diagnostic criteria for MM2C and MM2T‐type sCJD were proposed in 2020 and 2018 respectively, which has improved the rate of early clinical diagnosis of MM2‐type sCJD.^5^, ^16^

To this end, we reviewed our database of prion diseases collated over the past 10 years to estimate the proportion of probable MM2‐type sCJD in a Chinese cohort based on the clinical diagnostic criteria for MM2C and MM2T‐type sCJD. We have also summarized their clinical features and compared them with other types of sCJD to improve the early detection of MM2‐type sCJD. Our findings would enable patients and clinicians to the appropriate intervention.

## Methods

### Study design

Two hundred fifty‐four patients with suspected prion diseases admitted to the Department of Neurology at Xuanwu Hospital were consecutively recruited between February 1, 2012, and August 31, 2022. Patients admitted prior to January 2018 were retrospectively included and informed consent was waived by our independent ethics committee. Patients admitted as of February 2018 were prospectively included and informed consent was provided. For data collected retrospectively, telephone follow‐ups were conducted from February to April 2018 to determine disease progression and survival time, with regular follow‐ups for those patients still alive. Prospectively collected sCJD cases are followed up every 3 months at the clinic or by telephone, with the most recent follow‐up being in October 2022. Survival time was defined as the time period from the first symptomatic episode to death.

Patients with limited clinical features and no positive diagnostic test results as well as individuals with inadequate clinical features and a positive diagnostic test other than real‐time quaking‐induced conversion assay (RT‐QuIC); individuals with mutations in the *PRNP* or a clear family history of prion disease; or those diagnosed with possible Hashimoto's encephalopathy, viral or autoimmune encephalitis or paraneoplastic syndrome, etc.; or those lacking a comprehensive medical record; or clinical improvement or a clear diagnosis of another disease during the follow‐up period or loss to follow‐up were excluded. At the end of follow‐up, a total of 209 sCJD (88 retrospectively collected and 121 prospectively collected), 27 possible sCJD and 182 probable sCJD cases, who were diagnosed according to the updated clinical diagnostic criteria for CJD published in 2009^17^ and validated by the US Centers for Disease Control and Prevention (CDC) diagnostic criteria^13^ were included. Forty‐four probable MM2C‐type sCJD cases were diagnosed according to the criteria proposed by Hamaguchi et al in 2020.^5^ The diagnostic criteria for probable MM2C‐type sCJD included the following items: (1) progressive dementia. (2) no mutation and methionine homozygosity at codon 129 of *PRNP*. (3) hyperintensity lesions confined to the cerebral cortex on DWI on MRI of brain. (4) only one or none out of the following four clinical features within 6 months post‐onset: (i) myoclonus, (ii) pyramidal or extrapyramidal signs, (iii) cerebellar ataxia or visual impairment, and (iv) akinetic mutism. (5) in the absence of an alternative diagnosis from a routine investigation. Seven MM2T‐type sCJD cases were clinically diagnosed according to the criteria proposed by Cracco in 2018.^16^ The diagnostic criteria include: (A) symptoms (must meet 1–3): (1) cognitive impairment; (2) sleep disturbance and/or psychiatric symptoms; (3) two or more typical CJD symptoms: (i) ataxia or dysarthria, (ii) visual changes, (iii) pyramidal symptoms, (iv) extrapyramidal symptoms, (v) myoclonus. (B) diagnostic test results (one or more): (1) reduced sleep architecture (e.g., sleep spindles, K‐complexes) on polysomnography (PSG); (2) thalamic hypometabolism on brain Fluorodeoxyglucose‐positron emission tomography (FDG‐PET); (3) thalamic hypoperfusion on brain single photon emission computed tomography (SPECT). (C) lack of alternative etiology. (D) negative for *PRNP* mutation. The remaining 158 patients who do not meet the above two diagnostic criteria are classified as other types of sCJD (Fig. S1).

### Clinical and laboratory data

The following demographic and clinical variables were extracted from medical information and clinical records or by interview at baseline: age at onset (years), gender, duration of symptoms (months), initial symptoms, neurological manifestations during the clinical course, and auxiliary examination results [periodic sharp wave complexes (PSWCs) on electroencephalogram (EEG), PSG, cerebrospinal fluid (CSF) 14‐3‐3 protein, total‐tau protein and CSF/skin RT‐QuIC, codon 129 genotype on *PRNP*, structural neuroimaging and radionuclide neuroimaging, including SPECT and FDG‐PET].

### Laboratory methods

#### Genetic analyses

Genomic DNA was extracted from fresh peripheral blood leukocytes, and whole exome sequencing (WES) libraries were generated using the Agilent SureSelect Human All Exon V6 Kit (Agilent Technologies, Santa Clara, CA, United States). The detailed procedure has been described in our previous study.^18^

#### CSF 14‐3‐3 protein level detection and RT‐QuIC assay

As previously mentioned, CSF 14‐3‐3 protein detection and CSF/skin RT‐QuIC test were carried out at the Chinese CDC National Reference Laboratory for Human Prion Diseases.^19^, ^20^ In this study, skin samples measuring about 3 mm^2^ were taken from four different areas of the body (behind the ear, inner arm, inner thigh, and lower back) using a disposable skin biopsy punch manufactured by Acuderm Inc. in Fort Lauderdale, FL, USA. Three skin samples were taken from each patient. Normally, skin biopsies include the epidermis, dermis, and adipose tissue. The samples were then homogenized in a lysis buffer and stored at −80°C for future use. CSF and skin RT‐QuIC tests were performed on the same patients, but if the patient refuses lumbar puncture or has contraindications to lumbar puncture, only the skin RT‐QuIC test was performed.

The detailed steps for RT‐QuIC analysis have been described previously.^20^ The assay involved a reaction mixture containing rHaPrP90‐231, PBS, NaCl, EDTA, ThT, and SDS, along with CSF samples or diluted skin homogenates. ThT fluorescence was measured every 45 min, and a sample was considered positive if two or more wells showed positive reaction curves. The cutoff value was set as the mean value of the negative controls plus 10 times the standard deviation. The positive control was scrapie‐infected hamster brain homogenate, while the negative control was normal hamster brain homogenate.

#### Tau level test

Total tau protein levels in the CSF samples were measured by enzyme‐linked immunosorbent assay (ELISA) (Innotest hTAU‐Ag; Fujirebio, Belgium). A tau level higher than 1250 pg/mL was considered positive based on a previous study.^21^

#### Electroencephalogram

The CJD subjects received a 2 h EEG using 21 lead electroencephalographic transducer (Micromed, Italy). The EEG electrodes were placed according to the International 10–20 system. PSWCs were defined according to the criteria for PSWCs published in 1996.^22^

#### PSG and hypnogram analysis

A standard video PSG (E‐Series, Compumedics Ltd, Abbotsford, Australia) was performed. Sleep‐related laryngeal ringing, sleep breath disturbance (assessed by apnea‐hypopnea index), and involuntary movements were recorded. The sleep stages were scored offline according to the American Academy of Sleep Medicine scoring scale.^23^

#### Neuroimaging

All MRIs were performed at 3.0 T (Erlangen, Germany) with the following sequences: T1 weighted image, T2 weighted image, fluid‐attenuated inversion recovery (FLAIR), diffusion‐weighted imaging (DWI), and diffusion coefficient values. Abnormal or normal signal intensity was assessed using DWI and T2 FLAIR in each of the following regions: cortex, basal ganglia, and thalamus. SPECT scans were conducted using a double‐head rotating gamma camera (Siemens Healthcare, Erlangen, Germany) with ^99m^Tc‐ECD (25 mCi) to detect blood flow perfusion. PET scans were conducted using a GE Signa PET/MR 3.0 Tesla scanner (GE Healthcare, Milwaukee, WI) using ^18^F‐FDG FDG (~308 MBq) to track glucose metabolism in the brain.

### Statistical analysis

SPSS version 22.0 was used to conduct statistical analysis (IBM, Armonk, NY, United States). Continuous data are represented as the median (interquartile range) and compared using Mann–Whitney *U* test. Dichotomous data as percentages and compared using *χ* ^2^ test or Fisher test. A two‐tailed *p*‐value ≤0.05 was considered statistically significant when comparing the two groups. A Bonferroni adjustment was used to correct for multiple comparisons with the threshold for significance at 0.025 (two‐sided).

## Results

### The clinical features of clinically diagnosed MM2C‐type sCJD

**Figure 1:**
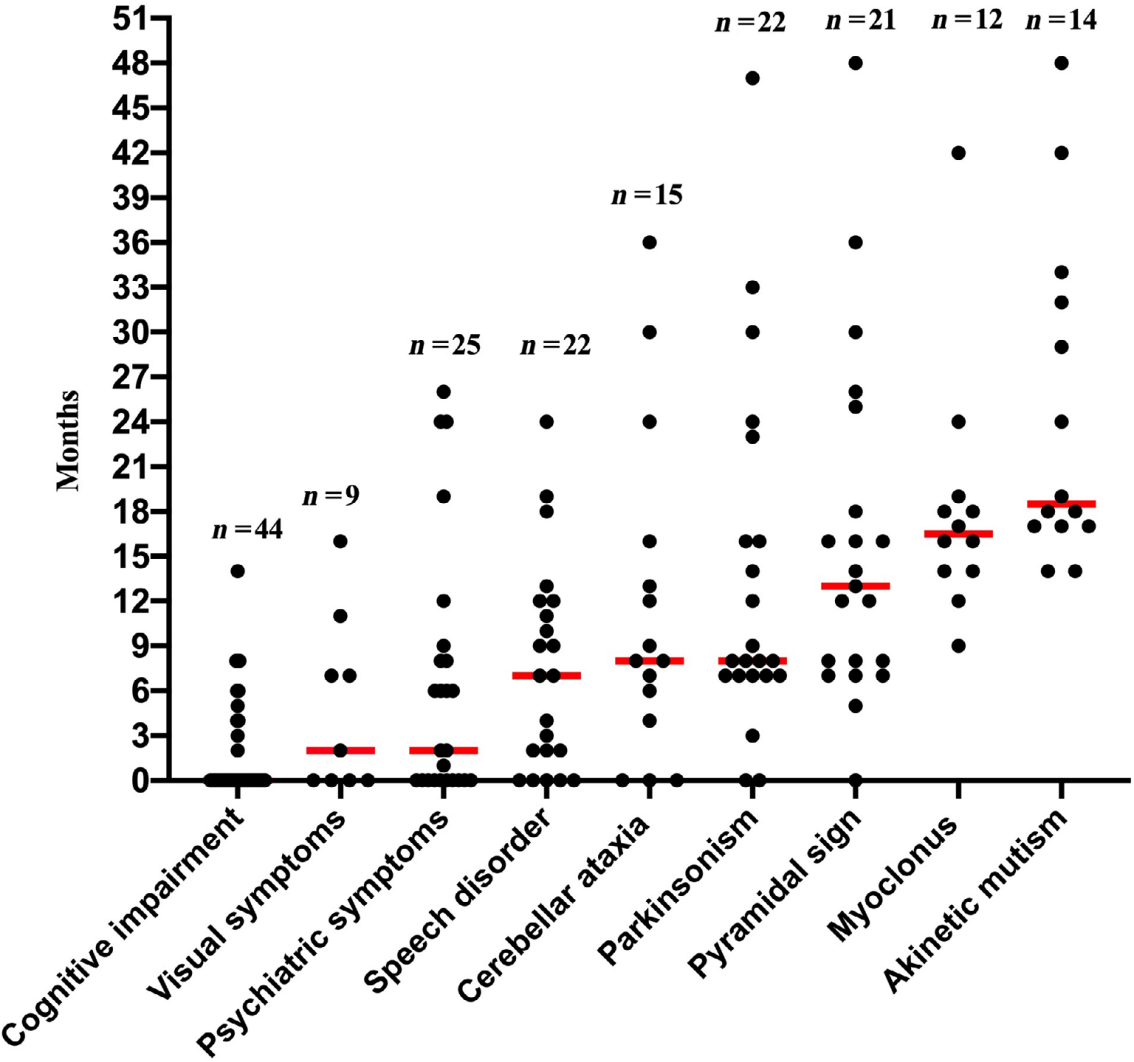
Scatter plot of the time (in months) to presentation of signs/symptoms in probable MM2C‐type sCJD patients. The red line indicates the median time.

A total of 44 patients (24 women and 20 men) with probable MM2C‐type sCJD were included and their clinical characteristics are summarized in Table S1. The median age at onset was 62.5 years (range: 43.0–76.0 years) and the median survival duration was 25.0 months (range: 11.0–82.0 months). Cognitive impairment was the most common first symptom and was presented in approximately 65.9% of the cases. Dizziness, numbness, or weakness of the limbs were the first symptoms in over 15% of the patients. Fourteen cases experienced one of the following symptoms within 6 months of the onset: myoclonus, pyramidal or extrapyramidal signs, cerebellar ataxia or visual impairment, or akinetic mutism. Furthermore, 5 of these patients presented with visual disturbance, 4 with cerebellar ataxia, 3 with extrapyramidal signs and 2 with pyramidal signs. The remaining 30 cases had no such symptoms within 6 months of onset. The process of symptom derivation is shown in Figure 1, with cognitive impairment, visual impairment, psychiatric symptoms, and language impairment mostly appearing within 6 months of onset, while other symptoms such as extrapyramidal symptoms, extrapyramidal, and myoclonus tend to appear after 7 months of onset.

Twenty‐seven patients underwent an MRI within 6 months of onset and 17 had an MRI within 3 months of onset. Hyperintensity lesions were detected in the cortical distribution of DWI in all cases. CSF RT‐QuIC test was conducted on 8 patients and seven of them tested positive, the skin RT‐QuIC test was conducted on nine patients, all of whom had positive results. RT‐QuIC was performed within 6 months in three cases and in one one case at 10 months after onset, when they lacked several typical clinical signs of sCJD and all results were positive.

In the absence of RT‐QuIC, 27 (61.3%) patients still could not meet the US CDC diagnostic criteria for possible sCJD at the time of admission to our hospital, even though the mean time from onset to admission was 6.0 months. Thirty died at follow‐up and 14 were still alive. All the patients could meet a probable diagnosis of sCJD at the end of the follow‐up.

### The clinical features of clinically diagnosed MM2T‐ type sCJD

**Figure 2:**
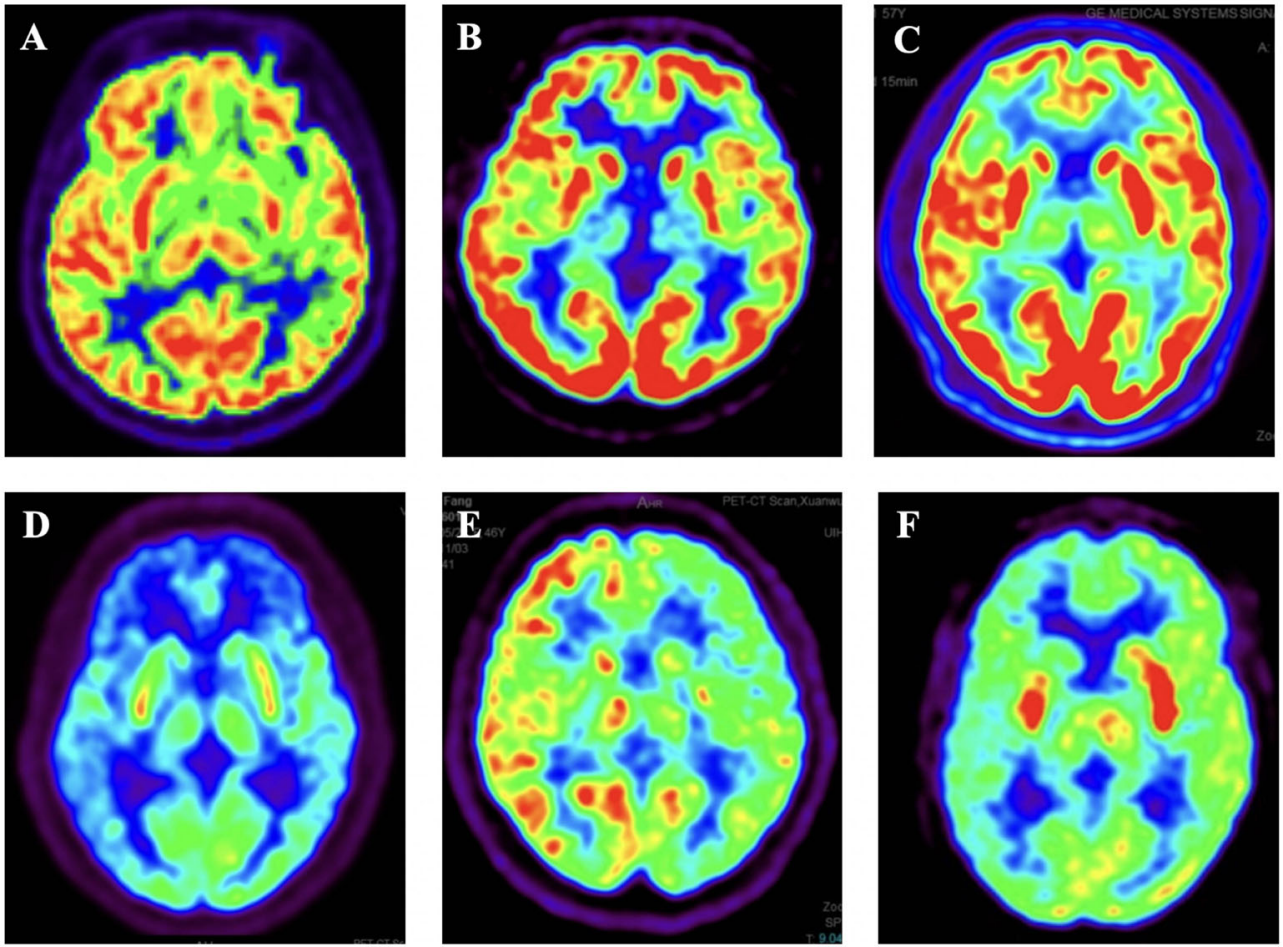
Thalamic metabolism in sCJD patients on PET. (A) An adult patient with a diagnosis of other types of sCJD, who underwent PET 1 week after the onset and had normal thalamic metabolism. Cases 2 (B) and 7 (C) were diagnosed with MM2T‐type sCJD and underwent PET 14 and 8 months after onset, respectively, both suggesting severe bilateral thalamic hypometabolism and relatively preserved cortical metabolism. Three other types of sCJD underwent PET 1，1 and 2 months after onset, respectively, suggesting bilateral (D), left (E), and right (F) thalamic hypometabolism with prominent cortical hypometabolism.

Due to the heterogeneity of the clinical features of MM2T‐type sCJD, and the lack of routine PET/SPECT and PSG for most patients with sCJD, we only included patients that presented with the symptoms of fatal familial insomnia (FFI) but lacked mutations in codon 178 of *PRNP*. Seven cases with a diagnosis of MM2T‐type sCJD were thus included, and their clinical characteristics are summarized in Table S2. All patients were male and the median age at onset was 52.0 years (range: 28.0–60.0 years) and the median survival duration was 23.0 months (range: 5.0–46.0 months). Six of the seven patients initially presented with sleep disturbances, and as the disease progressed, all patients developed the triad symptoms (cluster A + B + C). Organic insomnia and sleep‐related involuntary movements were the most frequent symptom in cluster A (100.0%), rapidly progressive dementia, psychiatric symptoms, and ataxia (100.0%) in cluster B, and weight loss (85.7%) in cluster C. Hyperintensity on DWI, PSWCs on EEG, positive for CSF 14‐3‐3 protein, CSF RT‐QuIC, and skin RT‐QuIC were positive in respectively 42.9%, 0%, 71.4%, 0%, and 100% of the patients. The proportion positive for PSG and thalamic hypometabolism on FDG‐PET was 100.0% and 80.0%. Patients with MM2T‐type sCJD have severe hypometabolism in bilateral thalamic but relatively preserved metabolism in the cerebral cortex (Fig. 2).

### Comparison of probable MM2C‐type sCJD, MM2T‐type sCJD with another type of sCJD

**Figure 3:**
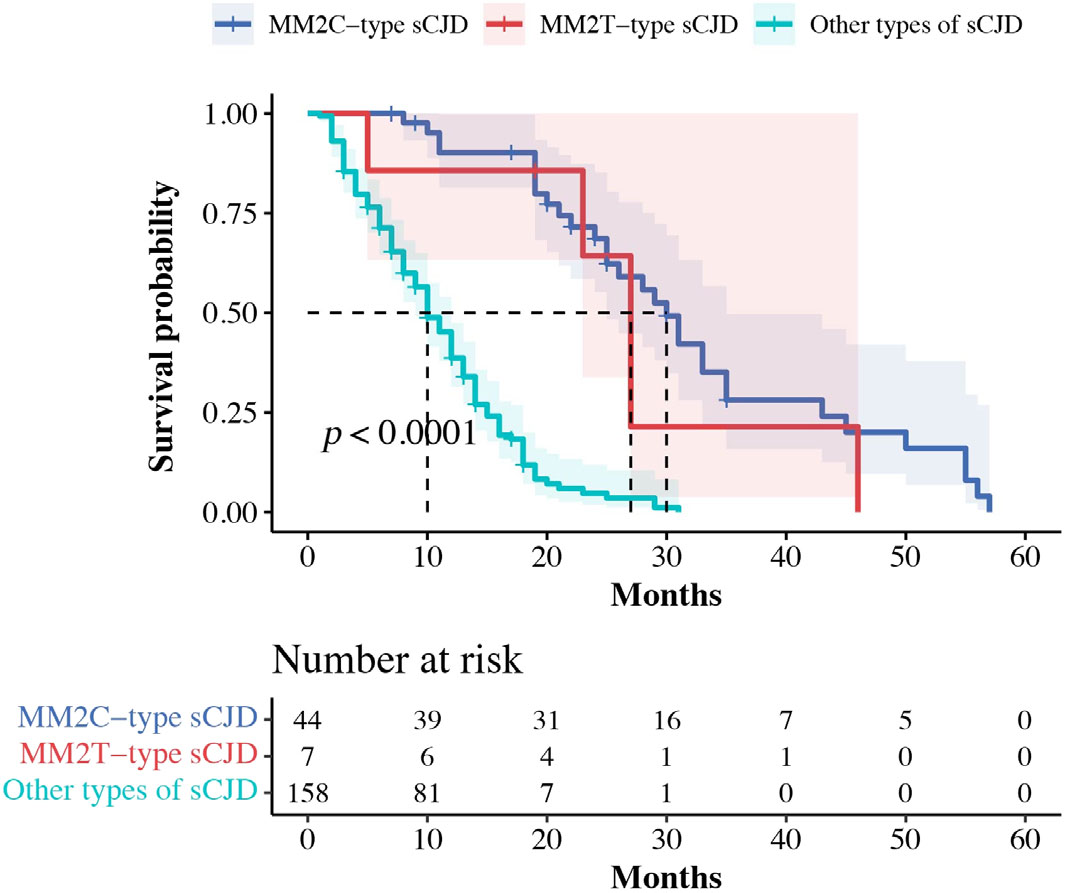
Survival Curves According to the clinical classification of sCJD.

A total of 158 patients had other types of sCJD, with a median age of onset of 62.0 years and a median survival duration of 10.0 months. These patients presented several typical features of sCJD early in the course of the disease. Compared to other types of sCJD, probable MM2C‐type sCJD had a longer disease duration, lower proportion of typical CJD symptoms, such as ataxia, myoclonus, visual symptoms, and mutism, higher hyperintensity on DWI and lower PSWCs on EEG. There was no significant difference between the two groups in terms of age at onset, gender, and first symptoms (Tables 1 and 2). Compared to other types of sCJD, clinically diagnosed MM2T‐type sCJD has a higher age of onset and longer disease duration, higher incidence of sleep disturbances as the first symptom, higher incidence of sleep, and autonomic symptoms during the disease course, lower hyperintensity on DWI, and PSWCs on EEG, and a higher incidence of thalamic hypometabolism/hypoperfusion on PET/SPECT, especially the bilateral thalamic (Table 2, Table S2, Figs 2 and 3).

**Table 1:** Comparison of the demographic data, clinical symptoms among probable MM2C‐type sCJD, MM2T‐type sCJD, and another type of sCJD.

	MM2C‐type sCJD (*N* = 44)	MM2T‐type sCJD (*N* = 7)	Other types of sCJD (*N* = 158)	*p* value
Age of onset, years, median (range)	62.5 (43.0, 76.0)	52.0 (28.0, 60.0)	62.0 (30.0, 83.0)*	0.009
Disease duration, months, median (range)	25.0 (11.0, 82.0)	23.0 (5.0, 46.0)	10.0 (1.0, 31.0)*	<0.001
Sex, male, %	20 (45.5)	7 (100.0)	87 (55.1)*	0.026
Initial symptoms, %				
Sleep‐related symptoms	1 (1.9)	6 (85.7)	1 (0.7)*	<0.001
Ataxia or gait disturbance	3 (6.8)	0	23 (14.6)	0.232
Psychiatric symptoms	8 (18.2)	2 (28.6)	16 (10.7)	0.151
Visual symptoms	0	0	19 (12.7)	0.034
Cognitive symptoms	30 (68.2)	0	70 (44.3)*	0.001
Dizziness/headache	4 (9.1)	0	29 (18.4)	0.167
Parkinsonism	2 (4.5)	0	6 (4.0)	0.843
Speech disorder	2 (4.5)	0	7 (4.4)	0.849
Limb's disturbance	3 (6.8)	0	16 (10.2)	0.544
Clinical manifestation				
Cognitive symptoms	52 (100.0)	7 (100.0)	158 (100.0)	1.000
Psychiatric symptoms	24 (54.5)	7 (100.0)	76 (48.1)*	0.024
Ataxia	15 (34.9)	7 (100.0)	99 (62.7)^#^, *	<0.001
Parkinsonism	22 (50.0)	4 (57.1)	108 (68.4)	0.143
Myoclonus	13 (29.5)	3 (42.9)	87 (55.1)^#^	0.011
Speech disorder	22 (50.0)	5 (71.4)	89 (56.3)	0.522
Visual symptoms	9 (20.5)	2 (28.6)	70 (44.3)^#^	0.014
Pyramidal sign	21 (47.7)	3 (42.9)	103 (65.2)	0.068
Akinetic mutism	14 (31.8)	1 (14.3)	86 (54.4)^#^, *	0.005

^#^ *p* < 0.025, MM2C‐type sCJD vs. other type of sCJD.

* *p* < 0.025, MM2T‐type sCJD vs. other type of sCJD.

**Table 2:** Comparisons of auxiliary examinations among probable MM2C‐type sCJD, MM2T‐type sCJD and other type of sCJD.

	MM2C‐type sCJD (*N* = 44)	MM2T‐type sCJD (*N* = 7)	Other types of sCJD (*N* = 158)	*p* value
Hyperintensity on DWI	44 (100.0)	3 (42.9)	135/157 (86.0)^#^, *	<0.001
Cerebral cortex	44 (100.0)	3 (42.9)	128/157 (81.5)^#^, *	<0.001
Basal ganglia	0	1 (14.3)	73/157 (46.5)^#^, *	<0.001
Thalamus	0	0	21/157 (13.4)^#^	0.023
PSWCs on EEG	9/43 (20.9)	0/7 (0)	69/137 (50.4)^#^, *	<0.001
Positive for CSF 14‐3‐3 protein	18/40 (45.0)	5/7 (71.4)	78/134 (58.2)	0.235
Positive for CSF Tau protein	2/3 (66.7)	0/2 (0)	7/9 (77.8)	0.115
Positive for CSF RT‐QuIC	7/8 (87.5)	0/2 (0)	17/23 (73.9)*	0.044
Positive for skin RT‐QuIC	9/9 (100.0)	2/2 (100)	26/27 (96.3)	0.811
PSG				
Reduced durations of REM	1/1 (100.0)	7/7 (100.0)	7/9 (77.8)	0.365
Sleep‐related involuntary movements	0	7/7 (100.0)	4/9 (44.4)	0.026
Sleep‐related dyspnea	0	7/7 (100.0)	7/9 (77.8)	0.043
Laryngeal stridor	0	2/7 (28.6)	0/9 (0)	0.198
Hypometabolism/hypoperfusion in cortex on PET/SPECT	17/18 (94.4)	5/5 (100.0)	42/43 (97.7)	0.734
Hypometabolism/hypoperfusion in thalamus on PET/SPECT	3/18 (16.7)	4/5 (80.0)	11/43 (25.6)*	0.017
Hypometabolism/hypoperfusion in bilateral thalamus on PET/SPECT	0	4/5 (80.0)	6/43 (14.0)*	<0.001
Codon 129 MM genotype	44 (100.0)	7 (100.0)	120/122 (98.4)	0.665

CSF, cerebrospinal fluid; DWI, diffusion‐weighted imaging; EEG, electroencephalogram; ND, not done; PET, positron emission tomography; PSWC, periodic sharp wave complex; REM, rapid eye movement; RT‐QuIC, real‐time quaking‐induced conversion test; SPECT, single photon emission computed tomography.

^#^ *p* < 0.025, MM2C‐type sCJD vs. other type of sCJD.

* *p* < 0.025, MM2T‐type sCJD vs. other type of sCJD.

## Discussion

In this study, we roughly assessed the proportion of probable MM2‐type sCJD in a Chinese cohort based on clinical diagnostic criteria and detailed the clinical features of probable MM2C‐type sCJD and MM2T‐type sCJD. Our findings suggest that a clinical diagnosis of MM2‐type sCJD can be made in approximately one‐quarter of patients and that the duration of the disease is usually longer in this group. In the early stages of probable MM2C‐type sCJD, typical clinical signs are often absent, but cortical hypersignal on DWI can be detected and RT‐QUIC can assist in early detection. Bilateral thalamic hypometabolism/hypoperfusion may help with the early identification of clinically diagnosed MM2T‐type sCJD cases. These results highlight the marked phenotypic heterogeneity of prion diseases and provide insights into the early identification of this rare disease.

MM2‐type sCJD has been inconsistently reported in Caucasian patients with sCJD, and the prevalence ranges from 4.5% to 13.6%.^4^, ^5^, ^6^, ^7^, ^8^ In this study, the proportion of probable MM2‐type sCJD was found to be 24.4%. This may be related to the fact that 98.5% of sCJD patients have the MM genotype at locus 129, which also implies a lack of MV1, MV2, VV1, and VV2 type sCJD cases in China, which constitute approximately 40–55% of the sCJD cases in the Caucasian population.^4^, ^5^, ^6^, ^7^, ^9^ Furthermore, the overall survival of Chinese sCJD patients is comparable to or longer than that of Caucasian patients.^24^, ^25^, ^26^, ^27^ Since MM1‐type sCJD is associated with significantly shorter survival compared to other forms of sCJD, the predominance of MM2‐type sCJD with a median survival of 12–36 months may contribute to the comparable or longer survival of Chinese sCJD patients. A previous study showed that Japanese sCJD patients generally have a longer disease course compared to Caucasians,^28^ which can be attributed to a longer survival period after reaching the akinetic mutism state, due to a higher rate of tube feeding.^29^ Differences in pathological types of sCJD may also influence the disease course, since 24.2% of the patients in Japan are diagnosed with MM2‐type sCJD or with a mix of MM2C pathological alterations.^5^

In line with previous studies, we found that probable MM2C‐type sCJD has a longer duration and lacks the typical clinical features of the disease in the early stages. This increases the frequency of misdiagnosis. However, hyperintensity confined to the cerebral cortex on DWI may contribute to the early diagnosis. In fact, abnormal DWI signals usually appear early in the course of the disease, even before the onset of symptoms.^30^ In addition, it has a high diagnostic specificity of over 90% for sCJD.^31^ Similar results were observed in type VV2‐type sCJD, second to MM(V)1 subtype in the Caucasian population, wherein 58 of 60 cases (96.7%) presented with DWI signal hyperintensity (especially in the striatum) within 3.7 ± 1.3 months after clinical onset in the absence of several typical clinical features.^15^ In patients who exhibit clinical manifestations that are likely due to sCJD, brain MRI is the most useful screening test, and it is typically readily available in hospitals due to its short acquisition time.^32^ The RT‐QuIC test represents a significant breakthrough in accurately diagnosing sCJD prior to death, with a sensitivity greater than 90% and a specificity approaching 100% in detecting CJD.^33^, ^34^ Implementation of this test may result in earlier and more precise diagnosis of CJD patients with a wider range of clinical signs and symptoms. In a study involving 214 confirmed cases of CJD and 50 CJD mimics, the combination of DWI on MRI and CSF RT‐QuIC enabled perfect classification of CJD from its mimics.^32^ Therefore, even if a patient does not have sufficient clinical indications for CJD, it is recommended to raise concerns for CJD and arrange for regular assessment if cortical hyperintensity is present on DWI after excluding other etiologies. To ensure early detection, optimizing RT‐QuIC (using CSF or other tissues such as skin) as soon as possible is advisable if circumstances permit. Due to the complexity of RT‐QuIC testing, which is still not routine in some countries and regions, the CSF total tau protein test represents an alternative method that has been shown to be more sensitive than CSF 14‐3‐3 protein and PSWCs on EEG.^33^, ^34^, ^35^

The clinical diagnosis of MM2T‐type sCJD considerably relies on PSG and SPECT/PET findings. However, we found that PSG may be less useful for the diagnosis of MM2T‐type sCJD since most patients with other types of CJD showed PSG abnormalities even in the absence of clinically significant sleep disturbances. This is consistent with Dai et al. who found that patients with sCJD often exhibit severe structural sleep disturbances by PSG, similar to patients with FFI.^36^ The main histological features of MM2T‐type sCJD are neuronal deficits and gliosis in the thalamic nuclei, and FDG‐PET and SPECT can directly detect metabolic or perfusion abnormalities in the thalamus. However, we detected thalamic hypometabolism/hypoperfusion in up to 25% of other kinds of sCJD. This shows that the specificity of SPECT/ FDG‐PET for MM2T‐type sCJD may not be as good as predicted. Furthermore, 75% (18/24) of patients with pathologically verified MM2T‐type sCJD had hypometabolism/hypoperfusion in the thalamus.^37^ Notably, the fact that all individuals with anomalies in thalamic metabolism or perfusion were bilateral, as was also seen in our investigation. This suggests that bilateral hypothalamic hypometabolism/hypoperfusion may contribute to the diagnosis of MM2T‐type sCJD. In addition, in patients with MM2T‐type sCJD, hypometabolism in the thalamus and cortex was more disproportionate and more pronounced in the thalamus, whereas in other types of sCJD, metabolism in the thalamus and cortex was more proportionally reduced.

This study has several limitations that should be taken into consideration. Firstly, all sCJD cases were diagnosed solely based on clinical symptoms without pathological evaluation due to laboratory conditions and ethical restrictions, which decreased the accuracy of the findings. Additionally, the cases included in this study were from a single Chinese center with a high proportion of MM at codon 129 of the *PRNP*, thus indicating the high potential for selection bias that ultimately limits generalization and application—particularly in patient populations with lower baseline prevalence of MM at codon 129 of the *PRNP*. Secondly, the criteria for MM2‐type sCJD have not been fully validated, and approximately one‐quarter to one‐third of cases of sporadic prion disease have mixed prion protein types (e.g., 1 and 2), which have significant effects on clinical phenotype. Thirdly, some participants did not undergo *PRNP* screening and were thus classified as other types of sCJD, since the majority of sCJD patients in China have *PRNP* codon 129MM genotype. This may have added some bias to the data. Fourthly, the diagnosis of all probable MM2C‐type sCJD in this study was primarily driven by MRI findings, while patients without typical MRI (11.1% as reported by Hamaguchi et al.^5^) were likely to have been overlooked or misdiagnosed and were not included in this series. Finally, a large number of patients lacked comprehensive CSF analysis, such as CSF total tau protein, due to lack of awareness and the relatively high cost of detection and self‐payment. In addition, only 18.2% of patients underwent RT‐QuIC analysis, as this test started relatively late in China and has not yet been fully popularized.

In conclusion, we have roughly described the proportion of clinically diagnosed MM2‐type sCJD in a Chinese cohort. In addition, we have summarized and compared the clinical data of probable MM2‐type sCJD with other types in order to aid its early diagnosis. Notably, it may not be possible to accurately identify the pathological form of sCJD based solely on the clinical characteristics and ancillary test results due to the clinical heterogeneity of sCJD. However, the current study makes a preliminary assessment of the sCJD pathological type before death, which can assist in predicting the prognosis of patients in advance.

## Author Contributions

Conceptualization, ZYC, and LYW; Data curation, ZYC, YK, MC, LL, KXX, and YC; Investigation, ZYC, YK, and JZ. Methodology, ZYC and YK, Validation, ZYC, JZ, and YK; Visualization, LYW; Writing—Original draft, ZYC; Writing—Review and editing, KY, JZ, MC, LL, KXX, YC, HY, JJL, LW, and LYW. All authors have read and agreed to the final version of the manuscript.

## Funding Information

This work was supported by the Ministry of Science and Technology of China (2019YFC0118600), National Natural Science Foundation of China (81971011) and Beijing Municipal Science and Technology Committee (D171100008217005, 7202060), the Xuanwu Hospital Science Program for Fostering Yong Scholars (QNPY2021001) and Beijing Postdoctoral Research Foundation (2022‐ZZ‐016).

## Conflict of Interest

None declared.

## Patient Consent for Publication

Written informed consent was obtained from each patient or their guardian.

## Supporting information


Figure S1.

Table S1.

Figure S1 Caption.

## Data Availability

The datasets used and analyzed during the current study are available from the corresponding author on reasonable request.
