# Supplementary figures and images for: Toward an early clinical diagnosis of MM2‐type sporadic Creutzfeldt–Jakob disease
Source: Ann Clin Transl Neurol. 2023 Jun 6;10(7):1209–18. doi: 10.1002/acn3.51802 (PMC10351658; doi:10.1002/acn3.51802)

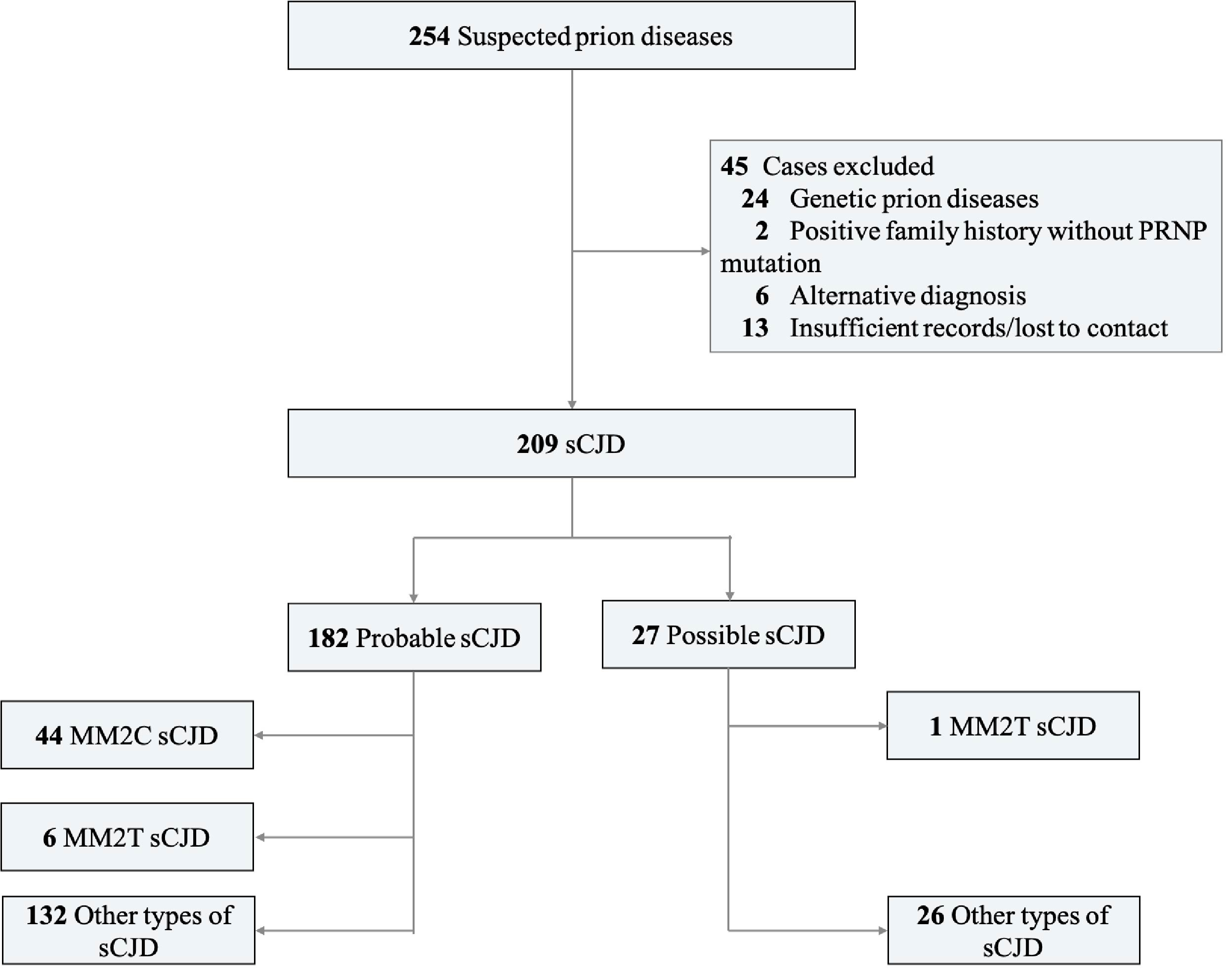

Supplement: Supplementary file 1 — Figure S1. [file ACN3-10-1209-s003.png]
