# Supplementary material for: Toward an early clinical diagnosis of MM2‐type sporadic Creutzfeldt–Jakob disease
Source: Ann Clin Transl Neurol. 2023 Jun 6;10(7):1209–18. doi: 10.1002/acn3.51802 (PMC10351658; doi:10.1002/acn3.51802)
Supplement: Supplementary file 2 — Table S1. [file ACN3-10-1209-s002.docx]

**Supplementary Table 1 The baseline data, clinical characteristics, and auxiliary findings for each probable MM2C-type sCJD patient**

| **Patient** | **Sex** | **Age at onset (Years)** | **Initial symptoms** | **Cognitive impairment (months)** | **Psychiatric symptoms (months)** | **Cerebellar ataxia (months)** | **Parkinsonism (months)** | **Myoclonus (months)** | **Speech disorder (months)** | **Visual impairment (months)** | **Pyramidal sign (months)** | **Akinetic mutism (months)** | **Hyperintensity lesions confined to the cerebral cortex on DWI (months)** |
| --- | --- | --- | --- | --- | --- | --- | --- | --- | --- | --- | --- | --- | --- |
| 1 | M | 56 | Cognitive impairment | Positive (0) | - | - | Positive (7) | - | - | Positive (2) | - | Positive (24) | Positive (6) |
| 2 | F | 57 | Dizziness, cognitive impairment | Positive (0) | - | - | - | Positive (12) | - | - | - | Positive (48) | Positive (6) |
| 3 | F | 57 | Dizziness, headache | Positive (0) | - | Positive (13) | - | - | Positive (12) | - | Positive (14) | - | Positive (2) |
| 4 | M | 47 | Speech disorder | Positive (3) | Positive (0) | - | Positive (16) | - | Positive (12) | - | Positive (16) | - | Positive (10) |
| 5 | F | 72 | Sleep-related symptoms | Positive (4) | - | - | Positive (3) | - | Positive (3) | - | Positive (5) | Positive (32) | Positive (3) |
| 6 | M | 60 | Cognitive impairment | Positive (0) | - | - | - | Positive (18) | - | - | - | Positive (18) | Positive (0) |
| 7 | M | 59 | Cognitive impairment, psychiatric symptoms | Positive (0) | Positive (0) | - | Positive (7) | - | Positive (4) | - | Positive (7) | Positive (17) | Positive (3) |
| 8 | F | 65 | Cognitive impairment, psychiatric symptoms | Positive (0) | Positive (0) | Positive (9) | Positive (23) | Positive (42) | - | - | - | - | Positive (3) |
| 9 | M | 45 | Cognitive impairment | Positive (0) | Positive (6) | - | Positive (8) |  | Positive (0) | - | Positive (8) | - | Positive (0) |
| 10 | F | 64 | Ataxia or gait disturbance | Positive (0) | - | Positive (0) | - | - | Positive (2) | Positive (0) | - | - | Positive (1) |
| 11 | F | 66 | Cognitive impairment | Positive (0) | - | Positive (8) | - | - | - | - | Positive (16) | - | Positive (6) |
| 12 | M | 57 | Psychiatric symptoms | Positive (8) | Positive (0) | - | - | Positive (14) | - | - | - | Positive (17) | Positive (15) |
| 13 | M | 58 | Cognitive impairment, parkinsonism | Positive (0) | Positive (12) | - | Positive (0) | - | Positive (9) | - | Positive (12) | - | Positive (12) |
| 14 | F | 60 | Cognitive impairment | Positive (0) | Positive (0) | - | Positive (14) | - | Positive (2) | - | - | - | Positive (3) |
| 15 | M | 58 | Limb’s disturbance | Positive (14) | Positive (26) | - | - | Positive (14) | - | - | Positive (26) | Positive (14) | Positive (1) |
| 16 | F | 43 | Cognitive impairment, psychiatric symptoms | Positive (0) | Positive (0) | Positive (24) | - | - | Positive (0) | - | - | Positive (42) | Positive (11) |
| 17 | M | 66 | Cognitive impairment | Positive (0) | Positive (2) | Positive (16) | Positive (12) | - | Positive (11) | Positive (11) | - | Positive (29) | Positive (8) |
| 18 | M | 65 | Cognitive impairment | Positive (0) | Positive (19) | - | - | Positive (19) | Positive (19) | - | Positive (25) | - | Positive (25) |
| 19 | M | 65 | Cognitive impairment | Positive (0) | Positive (6) | - | Positive (7) | Positive (9) | - | - | - | - | Positive (0) |
| 20 | F | 62 | Ataxia or gait disturbance | Positive (8) | Positive (8) | Positive (0) | - | - | Positive (0) | - | Positive (12) | Positive (19) | Positive (10) |
| 21 | F | 66 | Dizziness, cognitive symptoms | Positive (0) | Positive (1) | - | - | Positive (17) | - | - | Positive (0) | - | Positive (0) |
| 22 | F | 67 | Cognitive impairment, psychiatric symptoms | Positive (0) | Positive (6) | - | Positive (9) | - | Positive (9) | Positive (7) | - | - | Positive (8) |
| 23 | M | 70 | Cognitive impairment, psychiatric symptoms | Positive (0) | Positive (0) | Positive (6) | - | Positive (16) | Positive (13) | Positive (16) | - | Positive (14) | Positive (14) |
| 24 | F | 76 | Speech disorder | Positive (0) | - | - | - | - | Positive (0) | - | - | Positive (17) | Positive (9) |
| 25 | M | 67 | Cognitive impairment | Positive (0) | - | - | - | - | - | - | - | Positive (18) | Positive (1) |
| 26 | M | 63 | Cognitive impairment | Positive (0) | Positive (24) | Positive (36) | Positive (33) | - | - | - | Positive (36) | - | Positive (36) |
| 27 | F | 64 | Limb’s disturbance | Positive (2) | - | - | - | - | Positive (2) | - | Positive (7) | - | Positive (6) |
| 28 | M | 48 | Cognitive impairment | Positive (0) | Positive (24) | Positive (30) | Positive (24) | Positive (24) | Positive (24) | - | Positive (30) | Positive (34) | Positive (12) |
| 29 | M | 60 | Cognitive impairment | Positive (0) | Positive (0) | - | - | - | - | Positive (0) | - | - | Positive (5) |
| 30 | F | 56 | Cognitive impairment | Positive (0) | - | - | Positive (8) | - | - | - | Positive (8) | - | Positive (3) |
| 31 | F | 73 | Cognitive impairment | Positive (0) | - | - | Positive (47) | - | - | Positive (0) | Positive (48) | - | Positive (0) |
| 32 | F | 55 | Cognitive impairment | Positive (0) | Positive (9) | - | Positive (30) | - | - | - | - | - | Positive (12) |
| 33 | F | 56 | Cognitive impairment | Positive (0) | - | - | Positive (8) | - | Positive (7) | - | - | - | Positive (10) |
| 34 | F | 70 | Cognitive impairment | Positive (0) | Positive (8) | Positive (8) | Positive (8) | - | Positive (10) | - | - | - | Positive (10) |
| 35 | F | 61 | Cognitive impairment | Positive (0) | - | - | - | - | - | - | - | - | Positive (4) |
| 36 | M | 68 | Cognitive impairment | Positive (0) | - | Positive (7) | Positive (7) | - | Positive (7) | - | Positive (13) | - | Positive (1) |
| 37 | M | 52 | Ataxia or gait disturbance | Positive (8) | - | Positive (0) | Positive (16) | Positive (16) | Positive (0) | - | Positive (16) | - | Positive (0) |
| 38 | M | 60 | Limb’s disturbance | Positive (6) | Positive (6) | Positive (4) | - | - | - | - | - | - | Positive (6) |
| 39 | F | 58 | Cognitive impairment | Positive (0) | Positive (2) | - | - | - | - | - | - | - | Positive (4) |
| 40 | F | 70 | Cognitive impairment | Positive (0) | - | - | - | - | - | Positive (7) | Positive (7) | - | Positive (0) |
| 41 | F | 69 | Parkinsonism | Positive (5) | - | - | Positive (0) | Positive (18) | Positive (18) | - | Positive (18) | - | Positive (8) |
| 42 | F | 75 | Psychiatric symptoms, cognitive impairment | Positive (0) | Positive (0) | Positive (12) | - | - | - | - | - | - | Positive (6) |
| 43 | M | 72 | Psychiatric symptoms | Positive (4) | Positive (0) | - | - | - | - | Positive (0) | - | - | Positive (6) |
| 44 | F | 72 | Headache | Positive (6) | - | - | Positive (7) | - | - | - | Positive (8) | - | Positive (8) |

**Table Continue**

| Patient | Hyperintensity lesions confined to the basal ganglia on DWI (months) | Hyperintensity lesions confined to the thalamuson DWI (months) | PSWCs on EEG (months) | CSF total tau protein (months) | CSF 14-3-3 protein (months) | CSF RT-QuIC (months) | Skin RT-QuIC (months) | Hypometabolism/hypoperfusion in cortex on PET/SPECT | Hypometabolism/hypoperfusion in basal ganglia on PET/SPECT | Hypometabolism/hypoperfusion in thalamus on PET/SPECT | Hypometabolism/hypoperfusion in bilateral thalamus on PET/SPECT | 129 MM genotype | Disease duration (months) |
| --- | --- | --- | --- | --- | --- | --- | --- | --- | --- | --- | --- | --- | --- |
| 1 | Negative (6) | Negative (6) | Positive (7) | ND | Negative (7) | ND | ND | ND | ND | ND | ND | + | 30 |
| 2 | Negative (6) | Negative (6) | Negative (6) | ND | Negative (6) | ND | ND | ND | ND | ND | ND | + | 55 |
| 3 | Negative (2) | Negative (2) | Negative (14) | ND | Positive (16) | ND | ND | ND | ND | ND | ND | + | 45 |
| 4 | Negative (10) | Negative (10) | Negative 15) | ND | Positive (15) | ND | ND | Positive (15) | Positive (15) | Positive (15) | Negative (15) | + | 35 |
| 5 | Negative (3) | Negative (3) | Negative (6) | ND | Negative (11) | ND | ND | ND | ND | ND | ND | + | 43 |
| 6 | Negative (12) | Negative (12) | Negative (12) | ND | Negative (12) | ND | ND | ND | ND | ND | ND | + | 24 |
| 7 | Negative (6) | Negative (6) | Positive (6) | ND | Negative (6) | ND | ND | ND | ND | ND | ND | + | 19 |
| 8 | Negative (4) | Negative (4) | Negative (4) | ND | Positive (4) | ND | ND | Positive (4) | Negative (4) | Negative (4) | Negative (4) | + | 57 |
| 9 | Negative (8) | Negative (8) | Negative (8) | ND | Positive (8) | ND | ND | Positive (8) | Negative (8) | Negative (8) | Negative (8) | + | 31 |
| 10 | Negative (7) | Negative (7) | Positive (6) | ND | Negative (6) | ND | ND | Negative (2) | Negative (2) | Positive (2) | Negative (2) | + | 26 |
| 11 | Negative (6) | Negative (6) | Negative (6) | ND | Negative (6) | ND | ND | ND | ND | ND | ND | + | 33 |
| 12 | Negative (15) | Negative (15) | Negative (15) | ND | Positive (15) | ND | ND | Positive (15) | Positive (15) | Negative (15) | Negative (15) | + | 29 |
| 13 | Negative (12) | Negative (12) | Negative (12) | ND | Positive (12) | ND | ND | ND | ND | ND | ND | + | 82 |
| 14 | Negative (4) | Negative (4) | Negative (5) | ND | Positive (5) | ND | ND | ND | ND | ND | ND | + | 50 |
| 15 | Negative (24) | Negative (24) | Negative (24) | ND | Positive (24) | ND | ND | ND | ND | ND | ND | + | 33 |
| 16 | Negative (12) | Negative (12) | Negative (11) | ND | Negative (8) | ND | ND | ND | ND | ND | ND | + | 55 |
| 17 | Negative (8) | Negative (8) | Positive (13) | ND | Positive (12) | ND | ND | ND | ND | ND | ND | + | 31 |
| 18 | Negative (25) | Negative (25) | Negative (26) | ND | Positive (26) | ND | ND | ND | ND | ND | ND | + | 35 |
| 19 | Negative (7) | Negative (7) | Negative (7) | ND | Negative (7) | ND | ND | ND | ND | ND | ND | + | 11 |
| 20 | Negative (12) | Negative (12) | Negative (10) | ND | Negative (10) | ND | ND | ND | ND | ND | ND | + | 22 |
| 21 | Negative (0) | Negative (0) | Negative (1) | ND | Positive (1) | ND | ND | ND | ND | ND | ND | + | 25 |
| 22 | Negative (9) | Negative (9) | Negative (9) | ND | Negative (9) | ND | ND | ND | ND | ND | ND | + | 19 |
| 23 | Negative (16) | Negative (16) | Negative (18) | ND | Positive (18) | ND | ND | Positive (18) | Positive (18) | Negative (18) | Negative (18) | + | 19 |
| 24 | Negative (11) | Negative (11) | Positive (10) | ND | Negative (9) | ND | ND | Positive (9) | Positive (9) | Negative (9) | Negative (9) | + | 19 |
| 25 | Negative (2) | Negative (2) | Negative (2) | ND | Negative (2) | ND | ND | Positive (2) | Negative (2) | Negative (2) | Negative (2) | + | 21 |
| 26 | Negative (36) | Negative (36) | Negative (36) | Positive (36) | Negative (36) | ND | ND | ND | ND | ND | ND | + | 56 |
| 27 | Negative (6) | Negative (6) | Negative (7) | ND | Negative (7) | ND | ND | ND | ND | ND | ND | + | >20 (Alive) |
| 28 | Negative (30) | Negative (30) | Negative (19) | ND | Positive (30) | Positive (30) | Positive (30) | Positive (30) | Positive (30) | Negative (30) | Negative (30) | + | 37 |
| 29 | Negative (6) | Negative (6) | Negative (6) | ND | Negative (6) | ND | ND | ND | ND | ND | ND | + | >22 (Alive) |
| 30 | Negative (3) | Negative (3) | Negative (3) | ND | ND | ND | ND | ND | ND | ND | ND | + | 11 |
| 31 | Negative (0) | Negative (0) | Negative (34) | ND | ND | ND | ND | ND | ND | ND | ND | + | 20 |
| 32 | Negative (12) | Negative (12) | Negative (14) | ND | Positive (14) | ND | ND | Positive (14) | Negative (14) | Negative (14) | Negative (14) | + | >35 (Alive) |
| 33 | Negative (10) | Negative (10) | Negative (10) | ND | Negative (10) | ND | Positive (10) | Positive (10) | Negative (10) | Negative (10) | Negative (10) | + | >24 (Alive) |
| 34 | Negative (10) | Negative (10) | Positive (10) | ND | Negative (10) | ND | ND | ND | ND | ND | ND | + | > 10 (Alive) |
| 35 | Negative (4) | Negative (4) | Negative (4) | Negative (4) | Negative (4) | ND | ND | Positive (4) | Negative (4) | Negative (4) | Negative (4) | + | >20 (Alive) |
| 36 | Negative (1) | Negative (1) | Positive (12) | ND | Positive (12) | Positive (13) | Positive (13) | Positive (12) | Negative (12) | Negative (12) | Negative (12) | + | 28 |
| 37 | Negative (14) | Negative (14) | Negative (14) | ND | Negative (14) | ND | ND | Positive (14) | Negative (14) | Negative (14) | Negative (14) | + | >30 (Alive) |
| 38 | Negative (7) | Negative (7) | Negative (6) | ND | Positive (6) | Positive (6) | Positive (6) | Positive (6) | Negative (6) | Negative (6) | Negative (6) | + | >17 (Alive) |
| 39 | Negative (4) | Negative (4) | ND | ND | Negative (6) | Positive (6) | Positive (6) | Positive (6) | Positive (6) | Positive (6) | Negative (6) | + | >9 (Alive) |
| 40 | Negative (5) | Negative (5) | Negative (5) | ND | Negative (7) | Positive (7) | Positive (7) | Positive (7) | Negative (7) | Negative (7) | Negative (7) | + | >9 (Alive) |
| 41 | Negative (18) | Negative (18) | Positive (16) | ND | ND | Positive (18) | Positive (18) | ND | ND | ND | ND | + | >20 （Alive) |
| 42 | Negative (6) | Negative (6) | Negative (1) | ND | ND | ND | ND | ND | ND | ND | ND | + | >25 (Alive) |
| 43 | Negative (6) | Negative (6) | Negative (6) | ND | Positive (6) | Positive (6) | Positive (6) | Positive (6) | Positive (6) | Negative (6) | Negative (6) | + | >7 (Alive) |
| 44 | Negative (8) | Negative (8) | Positive (8) | Positive (8) | Positive (8) | Negative (8) | Positive (8) | ND | ND | ND | ND | + | >9 (Alive) |

Months from the onset of the disease at the time point when each examination showed following neurological manifestations or results of investigations are indicated. CSF, cerebrospinal fluid; DWI, diffusion-weighted imaging; EEG, electroencephalogram; ND, not done; PET, positron emission tomography; PSWC, periodic sharp wave complex; REM, rapid eye movement; RT-QuIC, real-time quaking-induced conversion test

**Supplementary Table 2 The baseline data, clinical characteristics, and auxiliary findings for each MM2T-type sCJD patient**

|  | Case 1 | Case 2 | Case 3 | Case 4 | Case 5 | Case 6 | Case 7 | |
| --- | --- | --- | --- | --- | --- | --- | --- | --- |
| Age of onset, years | 30s | 60s | 20s | 40s | 50s | 50s | 50s | |
| Disease duration, months | 27 | 5 | 23 | 27 | 46 | 19(alive) | 19(alive) | |
| **Initial symptoms** |  |  |  |  |  |  |  | |
| Organic insomnia | + | + | + | + | + | - | - | |
| Sleep-related movement disorders | - | - | - | - | - | + | - | |
| Psychiatric symptoms | - | - | - | + | - | - | + | |
| **Cluster A (Sleep-related symptoms) (months)** | | | | | | | |  |
| Organic insomnia | +(0) | +(0) | +(0) | +(0) | +(0) | +(4) | +(6) | |
| Sleep-related involuntary movements | +(7) | +(3) | +(8) | +(1) | +(34) | +(0) | +(7) | |
| Daytime sleepiness | - | - | - | +(1) | +(34) | +(5) | +(7) | |
| Sleep-related dyspnea | - | - | +(13) | - | +(34) | +(4) | +(6) | |
| Laryngeal stridor | +(2) | - | - | +(13) | +(36) | - | - | |
| **Cluster B (Neuropsychiatric symptoms) (months)** | | | | | | | |  |
| Rapidly progressive dementia | +(4) | +(3) | +(5) | +(1) | +(36) | +(8) | +(6) | |
| Psychiatric symptoms | +(0) | +(2) | +(5) | +(0) | +(32) | +(8) | +(0) | |
| Ataxia | +(7) | +(3) | +(10) | +(2) | +(36) | +(8) | +(8) | |
| Parkinsonism | +(4) | +(3) | - | +(9) | - | - | +(7) | |
| Myoclonus | +(7) | +(4) | - |  | +(36) |  | - | |
| Speech disorder | +(7) | +(3) | +(7) | +(1) | - | +(8) | +(2) | |
| Bulbar syndrome | +(12) | +(4) | +(10) | +(4) | +(34) | +(8) | - | |
| Neuro-ophthalmological abnormalities | - | - | +(14) | +(16) | - | +(12) | - | |
| Visual symptoms | - | - | +(5) | - | - | - | +(4) | |
| Pyramidal sign | +(12) | - | - | - | +(36) | - | +(8) | |
| Mutism | - | +(4) | - | - | - | - | - | |
| **Cluster C (Autonomic symptoms) (months)** | | | | | | | |  |
| Hypertension | - | - | - | - | - | +(6) | - | |
| Weight loss | +(3) | +(4) | +(6) | +(4) | +(36) | - | +(4) | |
| Bowel and bladder dysfunction | +(11) | +(4) | - | +(11) | - | - | - | |
| Sweating | - | - | - | +(2) | +(32) | - | - | |
| Tachycardia | - | - | - | - | - | +(14) | - | |
| Irregular breathing | - | - | - | - | +(32) | - | - | |
| **Auxiliary examination(months)** | | | | | | | |  |
| Hyperintensity on DWI | +(9) | +(4) | -(14) | -(16) | -(36) | -(12) | +(8) | |
| PSWCs on EEG | -(12) | -(4) | -(14) | -(16) | -(36) | -(12) | -(8) | |
| Positive for CSF 14-3-3 protein | +(12) | +(4) | +(14) | +(16) | -(36) | +(12) | -(8) | |
| Positive for CSF Tau protein | ND | ND | ND | ND | ND | -(12)  676pg/ml | -(8)  453pg/ml | |
| Positive for CSF RT-QuIC | ND | ND | ND | ND | ND | - | - | |
| Positive for skin RT-QuIC | ND | ND | ND | ND | ND | + | + | |
| Polysomnography |  |  |  |  |  |  |  | |
| Reduced durations of REM | +(12) | +(4) | +(14) | +(16) | +(34) | +(12) | +(8) | |
| Sleep-related involuntary movements | +(12) | +(4) | +(14) | +(16) | +(34) | +(12) | +(8) | |
| Sleep-related dyspnea | +(12) | +(4) | +(14) | +(16) | +(34) | +(12) | +(8) | |
| Laryngeal stridor | +(12) | - | - | +(16) | - | - | - | |
| Hypometabolism in thalamus on PET | ND | ND | +(14) | +(8) | +(34) | -(12) | +(8) | |

Months from the onset of the disease at the time point when each examination showed following neurological manifestations or results of investigations are indicated. CSF, cerebrospinal fluid; DWI, diffusion-weighted imaging; EEG, electroencephalogram; ND, not done; PET, positron emission tomography; PSWC, periodic sharp wave complex; REM, rapid eye movement; RT-QuIC, real-time quaking-induced conversion test
